# Susceptibility of Mature *Staphylococcus* Biofilms to Chinese Herbal Decoction Sanhuang Jiedu: An *In Vitro* Study

**DOI:** 10.1155/2020/7473942

**Published:** 2020-09-28

**Authors:** Shaoe Zhang, Xiao Wang, Xiaotao Shi, Honglue Tan

**Affiliations:** Henan Orthopedic Institute, Henan Luoyang Orthopedic-Traumatological Hospital (Henan Orthopedic Hospital), Luoyang 471000, China

## Abstract

**Background:**

External socking and washing with the Chinese herbal Sanhuang Jiedu decoction (SHJD) can effectively control local limb infections with bone and implant exposure. However, the antibiofilm activities of this decoction *in vitro* have not yet been investigated. Therefore, the aim of this study was to examine the effects and characteristics of SHJD on the mature biofilms of multidrug-resistant *staphylococci* on a titanium surface.

**Methods:**

Biofilm-forming methicillin-resistant *Staphylococcus epidermidis ATCC* 35984 and *S. aureus ATCC* 43330, and non-biofilm-forming *S. epidermidis ATCC* 12228 were selected as the experimental strains. The mature biofilms were prepared on titanium surfaces. The five experimental groups were based on dilution concentrations (DC) of SHJD: the control group (biofilm incubated with 0.85% NaCl solution), the SHJD (DC:1/8) group (initial SHJD solution was diluted 1/8), the SHJD (DC:1/4) group, the SHJD (DC:1/2) group, and the SHJD (DC:1/1) group (initial SHJD solution). The effects of SHJD on the mature biofilms were observed with the bacterial spread plate method, crystal violet (CV) staining, scanning electron microscopy, and confocal laser scanning microscopy.

**Results:**

After culture in tryptic soy broth for 72 h, *ATCC* 43300 and *ATCC* 35984 produced mature biofilms and *ATCC* 12228 did not. The optical density value of *ATCC* 12228 was 0.11 ± 0.02, significantly lower than that of *ATCC* 35984 (0.42 ± 0.05) or *ATCC* 43300 (0.41 ± 0.03) (*P* < 0.05). The mature biofilms of *ATCC* 43300 and *ATCC* 35984 clearly disintegrated when incubated for 12–24 h with SHJD (DC:1/1) or SHJD (DC:1/2), showing only scattered bacterial adhesion. In the SHJD (DC:1/4) group, although many residual bacterial colonies still clustered together, presenting a biofilm structure, it was very looser than that in the SHJD (DC:1/8) group in which the biofilm was similar to that in the control group. For *ATCC* 12228, only colony adhesion was observed, and the number of colonies decreased as the concentration of SHJD or the culture period increased. The quantitative results for the bacterial spread plate and CV staining showed significant differences between the SHJD groups (*P* < 0.05).

**Conclusion:**

SHJD has antibiofilm activity against multidrug-resistant *Staphylococcus* strains. It weakens or disrupts already-formed mature biofilms on titanium surfaces in a concentration- and incubation time-dependent manner.

## 1. Introduction

The management of refractory orthopedic-implant-associated infections is still challenging for surgeons because bacterial biofilms can form [1]. Biofilms are communities of cells within a self-produced extracellular matrix and are the commonest form of microbial life [2]. In the presence of this matrix, bacteria are highly resistant to antibiotics, with minimum inhibitory concentrations up to 1,000-fold higher than those of their planktonic forms [3]. Pathogenic bacterial biofilms can successfully evade the host's immune system and cannot be phagocytized and eliminated by polymorphonuclear leukocytes [4]. Therefore, the inhibition and removal of a bacterial biofilm from an infected area is an important procedure in the treatment of orthopedic infections.

The long-term application of systemic antibiotics is necessary for acute and chronic orthopedic infections associated with mature biofilms, together with local wound debridement [5]. However, the clinical reality is that refractory infections are always caused by multidrug-resistant (MDR) *Staphylococcus epidermidis* or *S. aureus*, and after the clinical susceptibility of the bacterium involved to antibiotics, vancomycin is usually the treatment of choice for biofilm-associated infections [6]. Unfortunately, the widespread use of vancomycin has led to the emergence of vancomycin-resistant *Staphylococcus* strains [7]. Linezolid is a new type of antibacterial agent with broad-spectrum antibacterial activity against most common Gram-positive bacteria and has been very useful in the treatment of MDR *Staphylococcus* infections when vancomycin fails. However, safety concerns often limit its use [7]. Clinical reports have also indicated the existence of linezolomine-resistant *staphylococci* [8]. Therefore, based on the emergence of antibiotic resistance, adverse side effects, and a lack of new antibiotics, other treatment methods are urgently required for orthopedic-biofilm-related infections caused by MDR bacteria.

Traditional Chinese medicine (TCM) has been used in the Chinese population to treat various infectious diseases for a long time, and many preparations have proven therapeutic effects [9–14]. According to the TCM theory, the Chinese herbal decoction Sanhuang Jiedu (SHJD), which is composed of *Scutellaria baicalensis Georgi* (Huang Qin), *Coptidis rhizoma* (Huang Lian), *Cortex Phellodendri chinensis* (Huang Bai), and so on, has heat-clearing and detoxifying functions. Based on the TCM theory, acute infection belongs to the “excess-heat syndrome”; the treatment of infection should be focused on the “heat-clearing.” SHJD has the effect of clearing “excess-heat”; thus, its clinical application can control tissue infections. We have used this preparation to treat local limb infections with bone and implant exposure by local soaking and washing methods. The observation of a large number of cases has shown that local infections are significantly controlled and that the granulation tissue grows well, improving the condition of the wound tissue so that further treatment is possible [12–14]. However, there have been few *in vitro* studies of the antibiofilm activity of this preparation. Therefore, we used MDR *S. aureus* and *S. epidermidis* to investigate the effects and characteristics of SHJD on mature biofilms of these bacterial strains on a titanium surface.

## 2. Materials and Methods

### 2.1. Preparation of SHJD

The ingredients of SHJD decoction were purchased from Henan Luoyang Orthopedic–Traumatological Hospital (Luoyang, China) and included *Scutellaria baicalensis Georgi* (Huang Qin), *Coptidis rhizoma* (Huang Lian), and *Cortex Phellodendri chinensis* (Huang Bai) with 25 g of each dry herb, and *Sophora flavescens* (Ku Shen), *Lonicerae japonicae* (Jin Yin Hua), *Forsythiae suspensa* (Lian Qiao), *Taraxacum mongolicum Hand.-Mazz.*(Pu Gong Ying), *Semen persicae* (Tao Ren), *Boswellia sacra* (Ru Xiang), *Commiphora myrrha* (Mo Yao), and *Radix glycyrrhizae* (Gan Cao) with 20 g of each dry herb (Table 1). The aqueous extract of SHJD was prepared as follows. First, all the ingredients were placed into an earthenware pot containing 3000 mL of ultrapure water for about 30 min, boiled with a strong fire, and then with a gentler fire about 30 min until the mixture was reduced to 1000 mL. Second, the water extract was filtered through filter paper and evaporated under reduced pressure to a final concentration of 0.7 g/mL based on the equivalent amount of the crude dry herb. Third, the water extract was sterilized by filtration through a 0.22 *μ*m filter. It was then added to a sterile NaCl solution (0.85%) to a final SHJD concentration of 0.4 g/mL. The aqueous extract of SHJD was stored at −20°C until use.

### 2.2. Preparation of Bacterial Strains

Three bacterial strains were used in this study: biofilm-forming MDR *S. aureus* (*ATCC* 43300), methicillin-resistant *S. epidermidis* (*ATCC* 35984), and non-biofilm-forming methicillin-sensitive *S. epidermidis* (*ATCC* 12228). The strains were thawed rapidly, used to inoculate tryptic soy agar (TSA; Shanghai Biotech Co., Ltd., China), and cultured statically at 37°C for 24 h. A colony was picked from this agar and suspended in 10 mL of tryptic soy broth (TSB; BD Biosciences, Franklin Lakes, NJ) supplemented with 0.5% glucose in a 50 mL centrifuge tube, and incubated for 10 h at 37°C with agitation at 100 rpm. The aliquots (25 *μ*L) of this preculture were transferred into other sterile tubes containing 10 mL of TSB and incubated at 37°C for 14 h, which was sufficient to ensure that the strains were at the end of the exponential phase of growth [15]. The bacteria in the suspensions were harvested by centrifugation for 5 min at 1000 rpm (Sorvall TC6, Dulont, USA), and the precipitate was washed three times with 0.15 M phosphate-buffered saline (PBS) to remove the remaining TSB. The pellet was resuspended in sterile PBS to an optical density (OD) at 600 nm (OD_600_) of 0.490, measured with a Synergy HT multidetection microplate spectrophotometer (BioTek, Winooski, VT), which corresponded to 109 CFUs (colony-forming units)/mL.

### 2.3. Mature Biofilms on Titanium Surfaces

To prepare the mature biofilms, sterile titanium discs (14 mm in diameter and 1 mm thick) were placed into the wells of a 24-well plate (Costar3548, USA), with six wells allocated to each strain. Bacterial cells of strains *ATCC* 35984, *ATCC* 43300, and *ATCC* 12228 were diluted to a concentration of 10^6^ CFUs/mL in TSB, and 2 mL of each cell suspension was added to a well. After incubation for 72 h without agitation, the discs were rinsed three times with PBS to remove nonadherent cells and transferred to another sterile 24-well microtiter plate. The mature biofilms formed on the titanium surfaces were identified with crystal violet (CV) staining assay [2], confocal laser scanning microscopy (CLSM), and scanning electron microscope (SEM) [16].

### 2.4. Effect of SHJD Concentration on Mature Biofilms

The five experimental groups were based on dilution concentrations (DC) of SHJD: the control group (biofilm incubated with 0.85% NaCl solution only), the SHJD (DC:1/8) group (initial SHJD solution was diluted 1/8), the SHJD (DC:1/4) group (initial SHJD solution was diluted 1/4), the SHJD (DC:1/2) group (initial SHJD solution was diluted 1/2), and the SHJD (DC:1/1) group (initial SHJD solution). Mature bacterial biofilms were prepared on titanium surfaces as described above. For each strain, an aliquot of 2 mL of NaCl and NaCl containing a different concentration of SHJD was dispensed into each well of the 24-well microtiter plate containing a titanium disc covered with a mature bacterial biofilm, with six replicate wells for each group. The plates were incubated under aerobic conditions with a low shaking rate of 100 rpm for 24 h. After incubation, the culture medium containing any planktonic bacteria was carefully removed from each well. The discs were transferred into another fresh 24-well plate and gently washed three times with sterile PBS to remove any loosely adherent bacteria. The residual biofilms of each strain on the titanium surfaces were analyzed with the following methods.

#### 2.4.1. Crystal Violet (CV) Staining

The biofilms on the disc surfaces were analyzed with CV staining according to a previous report [2]. Glutaraldehyde solution (2.5%, 2 mL) was pipetted into each of the drained wells and allowed to stand for 10 min to fix the cells. The glutaraldehyde solution was then removed, and the wells were washed three times with PBS. The PBS was removed, and 2 mL of 0.1% (w/v) aqueous CV solution was added to each well and incubated for 20 min at room temperature. The CV solution was discarded, and the wells were washed three times with PBS and air-dried for 12 h in the dark. The quantity of the biofilm was analyzed by adding 2 mL of 30% acetic acid to each well to dissolve the dye from the adherent cells (biofilm) for 30 min. Then, 200 *μ*L of the dye solution was transferred into a 96-well microtiter plate, with the samples from one group assigned to a vertical row of the plate wells. The OD_492_ of each well was determined with a plate reader.

#### 2.4.2. Spread Plate Method

The assay was performed according to a previous report [16]. After incubation for 24 h in different culture media, each titanium disc whose surface was covered with biofilm was lightly washed with PBS and transferred to a 10 mL glass tube containing 1 mL of PBS. The tubes were then placed in an ultrasonic bath (B3500S-MT, Branson Ultrasonics Co., Shanghai, China), and the bacteria or the biofilm on the discs were dislodged by ultrasonication (5 min) at an operating power of 150 W and a frequency of 50 Hz. Ultrasonication was followed by rapid vortex mixing (Vortex Genie 2, Scientific Industries, Bohemia, NY, USA) at maximum power for 1 min to remove any bacteria that still adhered to the discs. This method is known to effectively remove biomaterial-adherent bacteria [17]. The vortexed solutions were serially diluted 10-fold, and the final three dilutions were plated in triplicate onto TSA and incubated at 37°C for 24 h. The TSA culture plates containing colonies were photographed.

#### 2.4.3. CLSM Assay

After the titanium discs were washed three times with PBS to remove any nonadherent bacteria, they were stained with 500 *μ*L of fluorescent dye (LIVE/DEAD BacLight Bacterial Viability Kit, Molecular Probes, L13152) in the dark at room temperature for 15 min and were then analyzed with CLSM (Leica TCS SP2; Leica Microsystems, Heidelberg, Germany). The LIVE/DEAD kit contains two kinds of fluorescent dye. Viable and nonviable cells can be distinguished under a fluorescence microscope because SYTO™ 9 dye causes viable bacteria with intact cell membranes to display green fluorescence, whereas propidium iodide causes nonviable bacteria with damaged membranes to display red fluorescence. Images were acquired from random positions on the disc surfaces.

### 2.5. Effect of Incubation Time on Mature Biofilm

To observe the effects of SHJD on the mature biofilms on titanium surfaces after different periods of incubation (0, 4, 8, 12, 16, 20, and 24 h), 2 mL of NaCl and NaCl containing a different concentration of SHJD were transferred into each well of a 24-well culture plate containing a titanium disc covered with mature biofilm. Six replicate wells were analyzed for each strain at each time point. The plates were placed under aerobic conditions with a low shaking rate (100 rpm) for 24 h. At the specified time points, the culture medium was removed from the wells, and the titanium discs were gently washed three times with PBS and transferred to another 24-well plate. The bacterial biofilms on the titanium surfaces were analyzed with the following methods.

#### 2.5.1. The Spread Plate Method

At each time point, the titanium discs whose surfaces were covered with a residual biofilm were transferred to a 10 mL glass tube containing 1 mL of PBS. The tubes were placed in an ultrasonic bath, and the bacteria or biofilm on the discs were dislodged as described above. The vortexed solutions were serially diluted 10-fold, plated on TSA, and incubated at 37°C for 24 h. The numbers of colonies formed by the surviving bacteria were counted, and the numbers of bacteria in the biofilms were calculated and expressed relative to the titanium surface area (CFUs/mm^2^). The number of bacteria at 0 h was used as the initial value for each test solution, and the numbers at 4, 8, 12, 16, 20, and 24 h were used as the values after different incubation periods. The antibiofilm efficiency of SHJD was calculated after different periods of incubation with Eq. (1), according to a previous report [18]:

(1) $$\begin{array}{c} \text{Antibiofilm efficiency }\left(\%\right)=\left(\left[\text{initial cell number}-\text{cell number after treatment}\right]/\text{initial cell number}\right)\times 100\% \end{array}$$

#### 2.5.2. SEM Assay

At each time point, the titanium discs whose surfaces were covered with biofilm were transferred into another 24-well plate and gently washed three times with PBS to remove any nonadherent bacteria. The discs were prepared for SEM with standard procedures. The biofilms on the titanium surfaces were fixed with 2.5% glutaraldehyde for 2 h at 4°C, washed three times with cacodylate buffer, and dehydrated through a graded series of ethanol solutions (25%, 50%, 75%, 95%, and 100%). The samples were then freeze dried, sputter coated with gold, and observed with SEM (Joel JSM-6310LV, JEOL Ltd, Tokyo, Japan).

### 2.6. Statistical Analysis

All the experiments were performed in triplicate. The results are presented as means ± standard deviations (SD). The results for each bacterial strain were tested with one-way analysis of variance (ANOVA) with Tukey's multiple comparison test. The differences observed between samples were considered to be significant at *P* < 0.05. All analyses were performed with the SPSS 19.0 analysis software (SPSS Inc., Chicago, IL, USA).

## 3. Results

### 3.1. Morphology of Mature Biofilm

**Figure 1:**
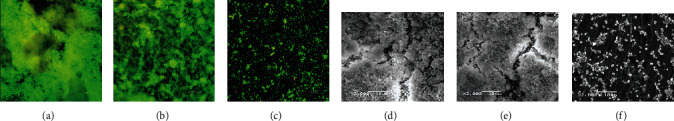
Mature biofilms on titanium surfaces after incubation for 24 h in TSB. (a) *ATCC* 43300 CLSM, (b) *ATCC* 35984 CLSM, (c) *ATCC* 12228 CLSM, (d) *ATCC* 43300 SEM, (e) *ATCC* 35984 SEM, and (f) *ATCC* 12228 SEM.

The CLSM images of the mature biofilms of each bacterial strain on the titanium surfaces are shown in Figure 1. After culture in TSB for 72 h, *ATCC* 43300 and *ATCC* 35984 produced numerous bacterial colonies, and the confluence of these colonies formed dense biofilms displaying high-intensity green fluorescence on the titanium surfaces (Figures 1(a) and 1(b)). In contrast, *ATCC* 12228 showed only scattered clustered colonies displaying low-intensity green fluorescence, indicating no biofilm formation (Figure 1(c)). SEM images showed that the biofilms of *ATCC* 43300 and *ATCC* 35984 were composed of many multilayered bacterial colonies, and the colonies clustered together to form dense structures (Figures 1(d) and 1(e)). However, the number and cluster size of the bacterial colonies of *ATCC* 12228 were dramatically lower than those of the other two strains, and the bacteria clustered as scattered colonies, suggesting that no mature biofilm formed (Figure 1(f)).

In the CV staining analysis, the OD_492_ value for *ATCC* 12228 was 0.11 ± 0.02, which was significantly lower than that for *ATCC* 35984 (0.42 ± 0.05) or *ATCC* 43300 (0.41 ± 0.03) (*P* < 0.05). The OD_492_ value for *ATCC* 12228 was <0.120, and those for *ATCC* 35984 and *ATCC* 43300 were both >0.240, suggesting that *ATCC* 12228 is a non-biofilm-producing strain and that both *ATCC* 35984 and *ATCC* 43300 are strong biofilm-producing strains [16, 19].

### 3.2. SHJD Weakens Mature Biofilms on Titanium Surfaces

#### 3.2.1. Quantitative Assay of CV Staining

**Figure 2:**
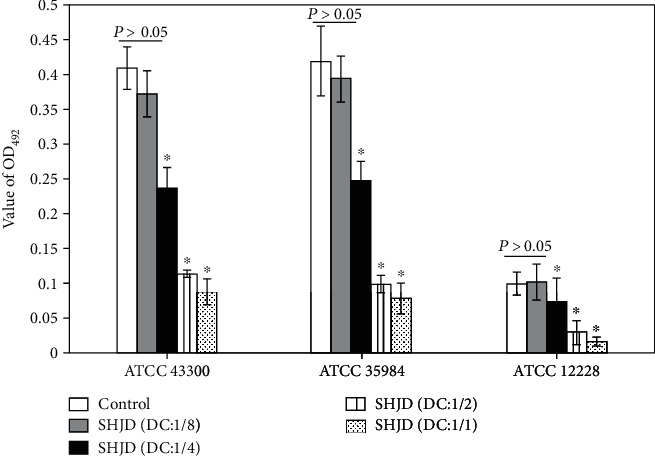
OD_492_ values for mature biofilms on titanium surfaces according to SHJD concentration. For each strain, ^∗^*P* < 0.05, compared with the control and SHJD (DC:1/8) groups.

As seen in Figure 2, the OD_492_ values for the SHJD (DC:1/4), SHJD (DC:1/2), and SHJD (DC:1/1) groups were significantly lower than those for the control and SHJD (DC:1/8) groups for all three bacterial strains (*P* < 0.05). In the control and SHJD (DC:1/8) groups, no significant differences in the OD_492_ values were detected (*P* > 0.05). However, the OD_492_ values differed significantly among the SHJD (DC:1/8), SHJD (DC:1/4), SHJD (DC:1/2), and SHJD (DC:1/1) groups (*P* < 0.05). For *ATCC* 12228, the OD_492_ value for each group was <0.120 because it is a non-biofilm-forming strain. However, for both strong biofilm-producing strains, *ATCC* 35984 and *ATCC* 43300, incubation with SHJD (DC:1/4), SHJD (DC:1/2), or SHJD (DC:1/1) caused a significant reduction in the OD_492_ value. The SHJD (DC:1/2) and SHJD (DC:1/1) groups had the lowest OD_492_ values (<0.120), and the SHJD (DC:1/4) group had the moderate values (<0.240) [19].

#### 3.2.2. Results of the Spread Plate Method

**Figure 3:**
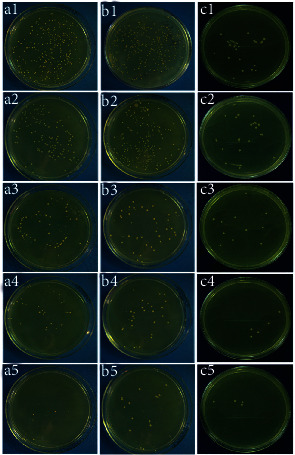
Images of bacterial growth after incubation on TSA for 24 h. (1) Control, (2) SHJD (DC:1/8), (3) SHJD (DC:1/4), (4) SHJD (DC:1/2), and (5) SHJD (DC:1/1) against (a) *ATCC* 43300, (b) *ATCC* 35984, and (c) *ATCC* 12228.

The colonies grown from the surviving bacteria in the residual biofilms on the titanium surfaces were counted on TSA. As shown in Figure 3, the number of viable bacterial colonies in the SHJD (DC:1/1), SHJD (DC:1/2), and SHJD (DC:1/4) groups were lower than those in the control and SHJD (DC:1/8) groups. As the concentration of SHJD decreased, the number of the surviving colonies clearly increased, indicating that the SHJD concentration dependently eradicated the mature biofilms of strains *ATCC* 43300 and *ATCC* 35984 and the adhesion of strain *ATCC* 12228 on a titanium surface.

#### 3.2.3. CLSM Assay

**Figure 4:**
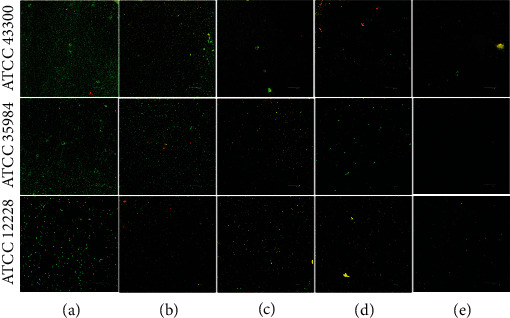
CLSM images of biofilms after incubation for 24 h. (a) Control, (b) SHJD (DC:1/8), (c) SHJD (DC:1/4), (d) SHJD (DC:1/2), and (e) SHJD (DC:1/1) groups.

As can be seen from Figure 4, the mature *ATCC* 43300 and *ATCC* 35984 biofilms on titanium surfaces displayed different fluorescence intensities on CLSM after they were incubated in different culture media. In the control and SHJD (DC:1/8) groups, the high-intensity green fluorescence on the titanium surfaces indicated that large numbers of bacterial colonies had accumulated and formed bacterial biofilms. However, the fluorescence intensity of the biofilms decreased in all the groups treated with SHJD. In the SHJD (DC:1/2) and SHJD (DC:1/1) groups, only scattered colonies displayed green fluorescence, indicating that the biofilms had been disrupted. In the SHJD (DC:1/4) group, the intensity of fluorescence was lower, suggesting the presence of some residual biofilm, although it was weakened. Only colony adhesion with scattered low-intensity green fluorescence was observed for strain *ATCC* 12228, because it is inherently unable to form a biofilm. In the SHJD-treated groups, the scattered green fluorescence decreased as the concentration of SHJD increased.

### 3.3. Different Incubation Times Affect Mature Biofilms

#### 3.3.1. Antibiofilm Assay

**Figure 5:**
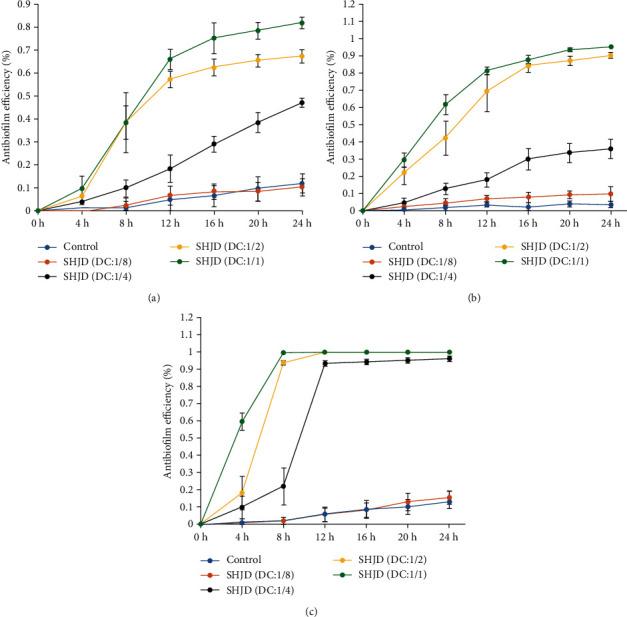
Effects of SHJD against mature biofilms after different periods of incubation. (a) *ATCC* 43300, (b) *ATCC* 35984, and (c) *ATCC* 12228. Among the SHJD-treated groups, SHJD exerted antibiofilm activity against *ATCC* 43300 and *ATCC* 34984, and antibacterial-adhesion activity against *ATCC* 12228.

The antibiofilm efficacy of SHJD, measured as the number of surviving bacterial colonies after different incubation times, was calculated with Equation (1) and is shown in Figure 5. These results show that the antibiofilm activities of SHJD at dilutions of 1/1 and 1/2 against each strain were significantly higher than those of the other dilutions at 8–24 h (*P* < 0.01). The antibiofilm efficacy of the initial SHJD solution diluted 1/4 was also higher than those of control solution and SHJD (DC:1/8) at 8–24 h (*P* < 0.05). From 12 to 24 h, no differences were observed between SHJD (DC:1/1) and SHJD (DC:1/2) against *ATCC* 35984, or among SHJD (DC:1/1), SHJD (DC:1/2), and SHJD (DC:1/4) against *ATCC* 12228 (*P* > 0.05). Significant differences were observed between SHJD (DC:1/1) and SHJD (DC:1/2) against *ATCC* 43300 (*P* < 0.05). There were no statistically significant differences in the antibiofilm efficacies of the control solution and SHJD (DC:1/8) against any strain at 4–24 h (*P* > 0.05). At 24 h, the antibacterial efficacy of SHJD (DC:1/2) against *ATCC* 43300, *ATCC* 35984, and *ATCC* 12228 was 67.3%, 90.3%, and 99.8%, respectively, and that of SHJD (DC:1/1) against *ATCC* 43300, *ATCC* 35984, and *ATCC* 12228 was 81.8%, 95.4%, and 99.9%, respectively.

#### 3.3.2. SEM Assay

**Figure 6:**
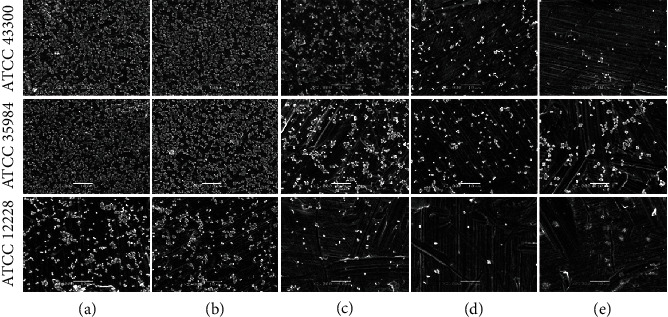
SEM images of biofilms after different periods of incubation with SHJD (DC:1/2). (a) 0 h, (b) 4 h, (c) 8 h, (d) 12 h, and (e) 24 h.

The SEM images in Figure 6 show the biofilm morphologies of the three bacterial strains in the SHJD (DC:1/2) group on the titanium surface after different periods of incubation. For strains *ATCC* 43300 and *ATCC* 35984, biofilms with dense multilayered bacterial colonies were observed at 0 and 4 h. At 8–24 h, the biofilms gradually disintegrated as the incubation period increased. The bacterial colonies on the titanium surfaces in the SHJD (DC:1/1) and (DC:1/2) groups were scattered and single, clearly indicating that the biofilm was disrupted. In the SHJD (DC:1/4) group, many residual bacterial colonies still clustered together, forming a moderate biofilm on the titanium surface. For strain *ATCC* 12228, only colony adhesion was observed after each period of incubation because it has no inherent biofilm-forming capacity.

## 4. Discussion

Infections caused by MDR *Staphylococcus* strains have now become a serious global public health concern because morbidity and the risk of mortality are increased in patients, and the economic burden has increased for health insurance systems [20, 21]. Studies have shown that 80% of clinical bacterial infections are caused by bacterial biofilms, including infections associated with medical devices, such as orthopedic implants, catheters, and heart valves [22]. The leading pathogens causing clinical orthopedic implant-related infections are *S. aureus* and *S. epidermidis* [22]. Biofilms are structured multicellular communities of sessile bacterial cells embedded within a self-produced matrix that is made up of proteins, DNA, and polysaccharides, which together act as physical and physiological barriers to antimicrobial agents [23]. The establishment of bacterial biofilms proceeds in two phases: the primary attachment of bacterial cells on a biomaterial is followed by the accumulation of bacteria in multiple layers and glycocalyx formation, generating a mature biofilm [5]. Despite many improvements in the management of orthopedic-biofilm-related infections, treatment failure still remains a major clinical problem because within mature biofilms, the bacteria are resistant to both antibiotics and the host immune system [24].

The long-term administration of effective systemic antibiotics is usually required to treat chronic orthopedic-biofilm-related infections [22]. However, the bacteria in biofilms are in a metabolically quiescent state, and the success of classical culturing to identify them may be as low as 30% [25]. Even if the biofilm bacteria are detected, a drug sensitivity test usually shows that the orthopedic-biofilm-related infection is caused by MDR *Staphylococcus*, for which vancomycin is the preferred treatment [6]. However, vancomycin administered systemically has poor bone permeability, with only 10% of the bone/serum ratio of the drug reaching the bone tissue, and an even smaller amount penetrating the bone tissue at the site of osteomyelitis [26]. Vancomycin kills bacteria by targeting their cell walls, whereas the bacteria in biofilms are protected by the biofilm membranes and are in an inactive and low metabolic state. This also greatly reduces the efficacy of vancomycin [27]. *In vitro* experiments have shown that vancomycin can penetrate biofilms and kill most (but not all) of the *staphylococci* growing in the biofilm within 24 h [28]. However, the concentrations tested in *in vitro* experiments range from 50 mg/L to 1000 mg/L, and it is not possible to achieve this concentration of the antibiotic with parenteral administration because vancomycin-induced nephrotoxicity can occur after conventional and high doses of the antibiotic [29]. Widespread abuse has also resulted in the emergence of a vancomycin-resistant strain of *Staphylococcus* [7]. Therefore, it is urgent that a new antibiofilm strategy be developed to overcome the drug resistance of bacteria and to combat the biofilm infections caused by the MDR bacteria. One of these strategies has been identified using a medicinal plant-derived product or herbal extract of TCM.

There is increasing interest in the use of medicinal plant-derived compounds as alternative antibacterial agents for infectious diseases [30–35]. However, most basic and clinical research has focused on the efficacy of the bioactive compounds isolated from plants against virulent pathogens, and few studies have examined the anti-infection activities of compound preparations [8–10, 32–35]. TCM has been used in the Chinese population to treat various infectious diseases for thousands of years, and many preparations have been shown to be therapeutically effective [8–10, 12–14]. The value of externally applied combination TCM preparations for the prevention and treatment of orthopedic infections has also been confirmed [12–14]. We have routinely used SHJD extract to directly soak and wash the infected wounds with bone and implant exposure in order to effectively destroy the bacterial biofilm and control the local infections of the extremities caused by multiple-antibiotic-resistant bacteria. The infected wound was soaked and washed for 30 min in SHJD extract twice a day, and after treatment for mean 3 weeks, we have found that local infections of the limbs are clearly controlled, the tissue swelling subsides, secretions and necrotic tissue are reduced, and the granulation tissue grows well, which provide better tissue conditions for wound healing. Modern medicinal research has shown that the heat-clearing and detoxifying herbs that comprise SHJD exert broad-spectrum antibacterial and bacteriostatic effects without bacterial resistance when they are applied in combination. *Semen persicae*, *Boswellia sacra*, and *Commiphora myrrha* promote blood circulation and the flow of qi, reduce swelling, and induce granulation, thus improving the local microcirculation and promoting tissue regeneration. The clinical application of SHJD has both antibacterial and anti-inflammatory roles, improving the local blood circulation to the wound and enhancing the local immunity, thus eliminating the infection and promoting wound healing [12–14].

To investigate the *in vitro* effects of the antibiofilm properties of SHJD in this study, the maturity of the biofilm was considered. Biofilms that have developed for only 24 h are always immature, and antibiotics can still kill the bacteria in these biofilms [36]. On the contrary, the metabolism of the bacteria in thick, multilayered, mature biofilms is relatively inactive, which allows them to tolerate extremely high antibiotic concentrations, so the eradication of these biofilms with antibiotics may be very difficult [36, 37]. Orthopedic implant-related infections are often detected after a few weeks in clinical practice, during which time the bacterial biofilm has developed into a mature form, with multiple layers and glycocalyx, and is therefore resistant to antibiotics. Based on these findings, we used a biofilm that had been incubated for 3 days to reflect the actual situation of mature biofilms [37]. *ATCC* 35984 and *ATCC* 43300 are powerful biofilm-forming strains, whereas *ATCC* 12228 is a non-biofilm-forming strain, and these were selected to test the weakening or eradication of mature biofilms by SHJD. We also examined the morphological changes in the biofilms that occurred under low-speed horizontal shaking, which simulated the possible flow of the tissue fluid in the infected wound. The quantitative and qualitative analyses in this study demonstrated that SHJD weakens and disrupts the mature biofilms formed by the antibiotic-resistant *Staphylococcus* on titanium surfaces. This effect tended to increase as either the concentration of SHJD or the period of incubation increased. Specifically, the mature biofilms of *ATCC* 43300 and *ATCC* 35984 on the titanium surfaces clearly disintegrated after incubation for 12–24 h with the initial SHJD solution (DC:1/1) or (DC:1/2), producing only scattered adherent bacterial colonies. With the SHJD (DC:1/4) treatment, although many residual bacterial colonies still clustered together, forming a weak biofilm on the titanium surfaces, the effect was greater than that in the SHJD (DC:1/8) group, in which the biofilm was only slightly weakened compared with that in the control group. Only colony adhesion was observed for strain *ATCC* 12228, but in the SHJD groups, the number of bacterial colonies decreased as the concentration of SHJD or the culture period increased.

Biofilm-related infections can be eradicated by antibiofilm agents that weaken or destroy the mature biofilm rendering cells to be susceptible to antibiotics [34]. In this study, the ability of SHJD to effectively weaken or disrupt mature biofilms suggests that it may increase the penetration of antibiotics in the biofilm and reduce bacteria antibiotic tolerance. This hypothesis needs to be validated by the synergistic effect of SHJD combined with conventional antibiotics on the mature bacterial biofilms. In the light of the findings of the current study, it is also important for further studies to clarify the mechanism of action of SHJD as an antibiofilm agent and to investigate the efficacy of SHJD to weaken the orthopedic-biofilm-related infections caused by MDR Staphylococcus *in vivo*.

## 5. Conclusions

In summary, this study demonstrates the SHJD decoction has antibiofilm activity against *Staphylococcus* strains with multidrug resistance, and it can weaken or disrupt already formed mature biofilms on the titanium surface with a dilution concentration-dependent and incubation time-dependent manner. It may be useful in the development of antibacterial agents to treat orthopedic-biofilm-related infections.

## Figures and Tables

**Table 1:** The composition of SHJD decoction.

Chinese name	Latin name	Part used	Proportion (g)
Huang Qin	*Scutellaria baicalensis Georgi*	Root	25
Huang Lian	*Coptidis rhizoma*	Root	25
Huang Bai	*Cortex Phellodendri chinensis*	Bark	25
Ku Shen	*Sophora flavescens*	Rhizome	20
Jin Yin Hua	*Lonicera Japonica*	Bud	20
Lian Qiao	*Forsythia suspensa*	Fruit	20
Pu Gong Ying	*Taraxacum mongolicum hand.-Mazz.*	Root	20
Tao Ren	*Semen persicae*	Seed	20
Ru Xiang	*Boswellia sacra*	Gummo-oleoresins	20
Mo Yao	*Commiphora myrrha*	Gummo-oleoresins	20
Gan Cao	*Radix glycyrrhizae*	Root	20

## Data Availability

The data used to support the findings of this study are available from the corresponding author upon request.

## Abbreviations

SHJD: Sanhuang Jiedu decoction
TCM: Traditional Chinese medicine
MIC: Minimum inhibitory concentration
TSB: Tryptic soy broth
TSA: Tryptone soy agar
CV: Crystal violet
SEM: Scanning electron microscopy
CLSM: Confocal laser scanning microscopy
MRSE: Multidrug-resistant *Staphylococcus epidermidis*
MRSA: Multidrug-resistant *Staphylococcus aureus*
MDR: Multidrug-resistance
Rpm: Revolutions per minute
CFUs: Colony-forming units
DC: Dilution concentrations
PBS: Phosphate-buffered saline
PI: Propidium iodide
OD: Optical density.
